# Emergence of Equine-like G3P[8] Rotavirus Strains Infecting Children in Venezuela

**DOI:** 10.3390/v17030410

**Published:** 2025-03-13

**Authors:** Esmeralda Vizzi, Rita E. Rosales, Oscar Piñeros, Rixio Fernández, David Inaty, Karolina López, Laura Peña, Angela De Freitas-Linares, Dianora Navarro, Sandra Neri, Osmary Durán, Ferdinando Liprandi

**Affiliations:** 1 Laboratorio de Biología de Virus, Instituto Venezolano de Investigaciones Científicas (IVIC), Caracas 1020-A, Venezuela; rosalesslab@gmail.com (R.E.R.); opineros2@gmail.com (O.P.); rixio.fernandez@gmail.com (R.F.); fliprand@gmail.com (F.L.); 2 Departamento de Pediatría, Clínica Las Ciencias, Caracas 1040, Venezuela; davidinatya@gmail.com; 3 Unidad de Gastroenterología y Nutrición, Hospital General “Dr. Miguel Pérez Carreño”, Caracas 1020, Venezuela; drakarolinalopezb@gmail.com (K.L.); dianora.navarro@gmail.com (D.N.); 4 Hospital de Niños “Dr. José Manuel de los Ríos”, Caracas 1050, Venezuela; laura25897@gmail.com (L.P.); angelajdefreitaslinares@gmail.com (A.D.F.-L.); dra.sandraneri@gmail.com (S.N.); 5 Hospital Militar Universitario “Dr. Carlos Arvelo”, Caracas 1020, Venezuela; osmaryduran25@gmail.com

**Keywords:** rotavirus, viral gastroenteritis, human equine-like G3 strains, G3 lineages, vaccines

## Abstract

*Rotavirus alphagastroenteritidis* is the leading cause of acute gastroenteritis worldwide in young humans and animals. In 2023–2024, a relatively high rotavirus detection rate (34.5%) was detected in children with diarrhea in Caracas. All rotavirus strains were typed as P[8], using a multiplex RT-PCR assay, while the G-type was not identified. This unusual pattern, not previously observed in Venezuela, prompted the VP7 gene sequencing of nineteen strains, which displayed a high sequence identity (99.3–100%) compatible with the G3 genotype. These strains clustered into a well-supported lineage IX encompassing human reassortants of equine-like G3P[8] strains described elsewhere, showing a very close genetic relationship (99.0–99.9%). Old G3 rotavirus isolates obtained from diarrheic samples in the past were included in the analysis and grouped into lineage I together with ancestral reference G3 strains. The novel G3P[8]s carry amino acid changes in VP7-neutralizing epitopes, compared with the RotaTeq-WI78-8-vaccine strain. Full genome sequencing of a representative strain revealed a genotype constellation including an equine-like G3P[8] in a DS-1-like backbone (I2–R2–C2–M2–A2–N2–T2–E2–H2), confirming the role of animal strains as a source of diversification, and the importance of unceasingly revising molecular typing strategies and vaccine efficacy to guarantee their success.

## 1. Introduction

*Rotavirus alphagastroenteritidis* is the most common cause of acute infectious diarrhea in humans and animals worldwide, which is associated with significant morbidity and mortality, particularly in developing countries. Approximately 30 to 50 percent of pediatric hospitalizations for diarrhea worldwide are attributed to rotavirus group A (RVA) infections [1]. In 2021, RVA caused 108,322 estimated deaths in children younger than five years old [2].

The implementation of RV vaccination has contributed to a reduction in diarrhea-related hospitalizations [3,4,5,6]. Nonetheless, evidence indicates that RV continues to be a health problem in several settings [6,7,8,9]. RVAs can also infect mammals such as pigs, calves, goats, lambs, horses, and avian species under natural conditions. Interspecies transmission is a common event, which contributes to their genetic diversification [10].

According to a binary classification system based on the capsid genes VP7 and VP4, RVAs can be classified into at least 42 G-types (VP7) and 58 P-types (VP4) (Rotavirus Classification Working Group—RCWG, https://rega.kuleuven.be/cev/viralmetagenomics/virus-classification/rcwg, accessed on 21 February 2025). Many combinations of G/P genotypes are feasible, but strain dominance varies by country, with G1P[8], G2P[4], G3P[8], G4P[8], and G9P[8] being the most prevalent in children with gastroenteritis also in the post-licensure period [11,12,13].

G1P[8], G9P[8], and G2P[4] were the most common genotypes from 1980 to 2019 worldwide [12,14]. G3P[8], G9P[8], and G2P[4] have been detected in similar proportions in Europe during the last few years [12]. G12P[8] has been an emergent rotavirus at various times after vaccine implementation in distinct countries [12,15,16,17], including Venezuela (Vizzi E, personal communication).

G3P[8], barely present and even absent during the 1990s worldwide, re-emerged in the following years in several world regions, such as Spain, Ethiopia, Italy, etc. [18,19,20]. G3 viruses are the most genetically heterogeneous rotaviruses described, which segregate into various lineages according to species-specific patterns, as described by Nishikawa et al., 1989 [21]. They infect humans and the widest range of animal species [10]. An earlier VP7 comparative analysis demonstrated that G3 porcine rotaviruses isolated in Venezuela in 1988–1989 were more closely related to human strains than to rotaviruses from other species, for which an exchange of genes between strains infecting the two species was suggested [22]. Occasionally, G3 strains of zoonotic origin can be transmitted from domestic animals, such as cats and dogs, to humans [10,20,23].

In 2008, a whole-genome classification was developed and adopted to describe circulating strains, based on the nomenclature Gx-P[x]-Ix-Rx-Cx-Mx-Ax-Nx-Tx-Ex-Hx, which represents the genotypes of every single protein, VP7-VP4-VP6-VP1-VP2-VP3-NSP1-NSP2-NSP3-NSP4-NSP5/6, respectively [24]. The majority of human RVAs are assigned to three genotype constellations: Wa-like (G1/3/4/9-P[8]-I1-R1-C1-M1-A1-N1-T1-E1-H1), DS-1-like (G2-P[4]-I2-R2-C2-M2-A2-N2-T2-E2-H2), and the less common AU-1-like (G3-P[9]-I3-R3-C3-M3-A3-N3-T3-E3-H3) [24]. Reassortment between RVA strains with different constellations is less common than within strains of the same constellation [25]. It remains a crucial mechanism for generating diversity that enhances the probability of immune evasion.

Inter-genogroup reassortants G3P[4] with a DS-1-like backbone [also named “equine-like G3” (EQL-G3) strains since they possess a VP7 gene of putative equine origin] were first reported in children from Japan [26]. DS1-like EQL G3P[8] strains possessing a novel constellation of genes have spread among human populations as an emergent pathogen since 2013 in Australia, Asia, Europe, the Caribbean, and North and South America, and have shown an endemic circulation in Australia and Brazil [27,28,29,30,31,32,33,34,35,36].

The first two rotavirus vaccines prequalified by the WHO in 2006 (Rotarix^®^ and RotaTeq^®^) have been introduced into the national immunization programs of 128 countries [International Vaccine Access Center (IVAC), https://view-hub.org/vaccine/rota, accessed on 24 February 2025]. The monovalent human RV vaccine Rotarix^®^ (GlaxoSmithKline Biologicals, Rixensart, Belgium) is based on the attenuated human G1P[8] RVA strain. The pentavalent human-bovine reassortant vaccine RotaTeq^®^ (Merck & Co., Inc., Whitestation, NJ, USA) contains human types G1, G2, G3, G4, and P[8] and bovine types G6 and P[5]. Both have demonstrated good safety and efficacy profiles in large clinical trials, mainly in the United States, Europe, and Latin America [37,38,39]. Two additional rotavirus vaccines were later approved and recommended for global use: ROTAVAC^®^ (monovalent G9P[11] strain, from Bharat Biotech International Ltd., Hyderabad, India), and ROTASIIL^®^ (pentavalent, human G1, G2, G3, G4, and G9 strains, and bovine G6P[5] strain, from Serum Institute of India, Pune, India) [40], both human–bovine vaccines. All these vaccines have demonstrated 90–95% protection against severe rotavirus gastroenteritis in countries with low mortality rates, and 44–70% in countries with high mortality rates [37,38,41]. The Lanzhou lamb rotavirus vaccine, manufactured by the Lanzhou Institute of Biomedical Products in China, and Rotavin-M1, manufactured by Polyvac in Vietnam, are in use in certain countries but are not available internationally [40].

Since their implementation in 2006, Rotarix^®^ and RotaTeq^®^ vaccines have been included in the Venezuelan national immunization program, with Rotarix^®^ being the vaccine of choice in the public healthcare sector, thus making it the most widely used. Rotavirus vaccine implementation in Venezuela determined a substantial reduction of 21% in rotavirus gastroenteritis hospitalizations [42]. However, information about the coverage rate in this country after 2017 has been lacking (EPI Country Report. Venezuela, 2020: https://www.paho.org/en/documents/epi-country-report-venezuela-2020, accessed on 28 January 2025) [43]. The wide heterogeneity of RVA infecting humans implies that licensed vaccines must protect against the diversity of strains in circulation. It is hypothesized that rotavirus vaccines may exert immunological pressures influencing the diversity of circulating strains [44], but no evidence supports such concerns [45]. Molecular surveillance is essential for detecting evolutionary changes that lead to the emergence of strains with disseminating potential.

The prevalence of G1P[8] and G2P[4] has varied among children with acute gastroenteritis in Venezuela over time [46,47]. G9P[8] strains were moderately circulating between 2003 and 2008, and unusual strains of probable animal origin such as G8P[14] have been occasionally described. The G3P[8] genotype was predominantly circulating in Venezuela between 2001 and 2005, but it was occasionally detected until 2008, when it disappeared [46,47].

This paper investigates the RVA infection rate among children in Caracas who recently experienced diarrhea. The study aims to analyze the genetic diversity of the circulating RVA strains in Venezuela using a conventional G-typing strategy and additional molecular tools. It also seeks to detect emerging viruses that may result from interspecies transmission and reassortment.

## 2. Materials and Methods

### 2.1. Samples

The study analyzed 58 stool samples from children less than 12 years old with acute gastroenteritis, collected between March 2023 and April 2024 at three health centers located in Caracas (Venezuela), Hospital de Niños “Dr. J.M. de los Ríos”, Hospital Militar Universitario “Dr. Carlos Arvelo”, and Clínica Las Ciencias, the first one being the biggest pediatric hospital in the Capital City. Acute gastroenteritis was defined as ≥3 looser-than-normal stools within a 24 h period, or an episode of forceful vomiting and any loose stool, and less than 7 days of duration.

The stool samples were collected within 12 h of ambulatory care or hospital admission and were stored at −20 °C until processing. Informed consent was obtained from the parents or legal guardians of each child. Ethical approval was granted by the Ethics Committee of the Instituto Venezolano de Investigaciones Científicas (IVIC).

### 2.2. Rotavirus Molecular Detection and G/P Genotyping

Viral DNA/RNA were extracted from 200 μL of clarified stool suspension (5% *w*/*v* in phosphate-buffered saline, pH 7.2) by Viral Nucleic Acid Extraction Kit II (Geneaid Biotech Ltd., New Taipei City, Taiwan) based on a spin-column procedure, according to the manufacturer’s instructions. The presence of the rotavirus genome was investigated using an in-house multiplex reverse transcription–polymerase chain reaction (RT-PCR) assay for gastroenteritis viruses (rotavirus, astrovirus, and calicivirus), as previously described [48], using an oligonucleotide primer pair able to amplify a 379 bp conserved region of the VP6 gene of rotavirus [49]. All PCR reactions were performed using 3 μL of cDNA in a final volume of 25 μL.

Rotavirus G (VP7) and P (VP4) genotypes were determined by semi-nested multiplex PCR with two rounds as described previously [46] with some modifications: in the first round, a pair of oligonucleotide primers for conserved regions of the genome of human rotaviruses were used to amplify genes VP7 and VP4, named VP7-F/VP7-R for G-typing [50] and A/B for P-typing [51]. In the second round, type-specific primers for VP7 [G1-4, G8-10, and G12 type] [46,52] and VP4 (P[4], P[6], P[8], P[9], and P[14] type) [46] were used.

All the PCR products were analyzed using agarose (FMC Bioproducts, Rockland, ME, USA) gel electrophoresis and ethidium bromide staining.

### 2.3. VP7 Gene Nucleotide Sequencing of RVA-Positive Samples

All the RVA-positive samples were investigated further by direct sequencing of the VP7 gene. Briefly, first-round VP7 gene PCR products were purified using the QIAquick^®^ PCR purification kit (QIAGEN^®^, Hilden, Germany); the amplicons obtained were processed in both directions using the BigDye™ Terminator v3.1 Cycle Sequencing Kit (Applied Biosystems™, Foster City, CA, USA), with the same primer set as the PCR, VP7-F and VP7-R, as previously described [46,50], and sequenced on the ABI 3130XL Genetic Analyzer (Applied Biosystems™, Foster City, CA, USA) at the Laboratorio de Genética Forense, Fundación Instituto de Estudios Avanzado (Caracas, Venezuela).

### 2.4. Historical Samples Included in the Study

Six old rotavirus strains previously identified as G3 type were randomly selected for VP7 gene sequencing (Table 1). This selection was part of a collection that included 1755 stool samples collected from 2003 to 2013, which were obtained from pediatric patients (<10 years old) admitted for acute diarrhea and treated at various health centers over time as part of a rotavirus diarrhea surveillance program. The cities of origin for the whole sample were Valencia (Carabobo State, 2003), Caracas (Capital City, 2007–2008), Maracay (Aragua State, 2011–2013), and Barcelona (Anzoátegui State, 2012–2013), Venezuela (Table 1). Rotavirus-positive samples had been identified by enzyme-linked immunosorbent assays, immunochromatographic tests, in-house multiplex RT-PCR (as described above), or a commercial PCR multiplex system (Seeplex^®^ diarrhea-V ACE detection assay, Seegene Inc., Seoul, Republic of Korea). One additional G3 rotavirus, identified in a previous study testing wastewater samples directly from domestic effluent discharge points from Caracas, and identified using an in-house multiplex RT-PCR [48], was also included in the VP7 gene analysis. All the rotavirus G-typing assays were performed by semi-nested multiplex RT-PCR, as described in Section 2.2.

### 2.5. Phylogenetic Analysis

VP7 nucleotide sequencing data from the rotavirus-positive samples included in the analysis were verified using the BioEdit Sequence Alignment Editor 7.2.5. The consensus sequences were compared with reference strains available in the NCBI GenBank database, using the Basic Local Alignment Search Tool (BLAST^®^) version BLASTN 2.16.1+ (https://blast.ncbi.nlm.nih.gov/Blast.cgi, accessed on 21 January 2025), and the genotype assignments were realized by Rotavirus A Genotyping Tool Version 0.1 (https://www.rivm.nl/mpf/typingtool/rotavirusa/, accessed on 21 January 2025), developed by the National Institute for Public Health and the Environment, Bilthoven, KU Leuven University (RIVM) and EMWEB (Belgium).

Multiple sequence alignment of VP7 nucleotide (nt) and deduced amino acid (aa) sequences was performed using the ClustalW method. Phylogenetic trees were built with the neighbor-joining method in MEGA (v7.0.26) software [54], using Kimura’s two-parameter substitution model and bootstrap testing using 1000 replicates. Sequence distances were calculated using the p-distance algorithm in MEGA7.

### 2.6. RT-PCR Specific for Equine-like G3 Rotavirus

An RT-PCR assay using specific equine-like G3 VP7 primers, EQG3FWD 5′-CTGCATACGCTAATTCTACACAAGG-3′ (nt 242–266) and EQG3REV 5′-GATCGTACAAGTAGCCGTAGTAAC-3′ (nt 786–763), was also employed to amplify G-not-typed (GNT) samples, as previously described [27].

### 2.7. RVA Full-Genome Sequencing

To understand the genetic background of the RVA found in Venezuela and its relatedness to other RVA strains globally, full-genome analysis was performed for one randomly selected rotavirus strain obtained from Caracas, labeled 3-CLC, and isolated in 2023 during this study. For this purpose, each gene segment was partially amplified from cDNA with the specific primers described in the Supplementary Materials (Table S1). The purified PCR products were automatically analyzed by SANGER by DNA amplification in both directions with the PCR primers described above, using the BigDye Terminator labeling method (Macrogen^®^ Inc., Seoul, Republic of Korea).

### 2.8. Nucleotide Sequences

The nucleotide sequences obtained in this study have been deposited in the GenBank under the accession numbers PV036887-PV036921.

### 2.9. Synonymous and Non-Synonymous Change Analysis

The VP7 nucleotide sequences obtained in the present study were compared with a reference strain by the Synonymous Non-synonymous Analysis Program (SNAP v2.1.1) (https://www.hiv.lanl.gov/content/sequence/SNAP/SNAP.html, accessed on 23 January 2025) [55]. Highlighter software enables codon-aligned nucleotide sequences to be compared with one query master sequence and highlights silent and non-silent mutations. Furthermore, it calculates synonymous and non-synonymous substitution rates based on a set of codon-aligned nucleotide sequences.

### 2.10. Statistical Analysis

The data were analyzed by EpiInfo™ software (version 7.2.6, CDC, Atlanta, GA, USA) for variable comparisons, using 2 × 2 tables with the χ^2^ test or Fisher’s exact test (two-tailed) when the size sample was less than 5. Student’s test was applied to compare ages. A *p*-value < 0.05 was considered statistically significant.

## 3. Results

### 3.1. Rotavirus Prevalence and G/P Genotype Assignment

From March 2023 to April 2024, 20 (34.5%) of the 58 stool samples analyzed from children with diarrhea tested positive for RVA using the multiplex RT-PCR assay for gastroenteritis viruses. All rotavirus-positive samples were from symptomatic children living in urban settings. The median age of children infected with RVA was calculated to be 13 months. Most of the children (75%) were unvaccinated, due to limited access to vaccines. No significant differences were observed in age or rotavirus vaccination status between RVA-positive and -negative children (*p* > 0.05).

None of the 20 positive rotavirus strains could be successfully G-typed (GNT) using the semi-nested multiplex PCR protocol, as some were misclassified as G9 or showed mixed undefined electrophoretic patterns. On the other hand, VP4 typing successfully determined the P-type, identifying all 20 strains as P[8].

### 3.2. VP7 Sequence Analysis of Venezuelan RVA Strains

Partial nucleotide (nt) sequences of the VP7 gene were obtained from 19 out of 20 GNTP[8] RVA strains circulating in Venezuela during the 2023–2024 season, as well as from 6 old strains collected between 2003 and 2008 (Table 1). The verified consensus sequences ranged from 542 to 854 base pairs (bp).

All Venezuelan VP7 nucleotide sequences obtained from both groups exhibited at least 80% similarity with the G3 genotype of rotaviruses, as determined by the Rotavirus A Genotyping Tool. BLAST searches of the sequenced amplicons confirmed the G3 genotype assignment and anticipated a closeness of the 2023–2024 GNTP[8] strains to rotaviruses of human–animal-like origin.

The RT-PCR assay conducted on these samples using equine-like G3 VP7-specific primers successfully yielded a 544 bp fragment corresponding to the amplification of the VP7 gene, as expected [27]. This result enabled the accurate characterization of the strains as equine-like G3P[8].

**Figure 1:**
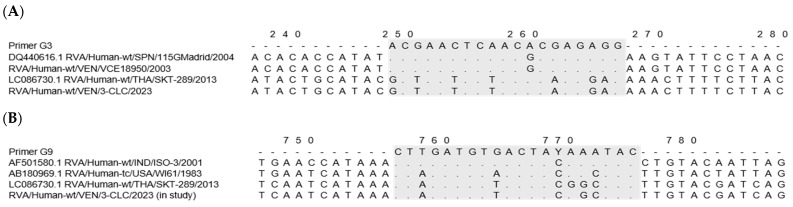
Multiple alignments of the VP7 rotavirus nucleotide sequences reveal mismatches at the primer-binding region (in gray) used in the G-typing assay [56]. (**A**) The G3 primer sequence was compared to G3 reference strains (SPN/115Madrid and equine-like/THA/SKT-289), and Venezuelan strains: VEN/VCE18950/2003 (old G3), and VEN/3-CLC/2023 (novel equine-like G3). (**B**) The G9 primer sequence was compared to G9 reference strains (IND/ISO-3, and the ancestral USA/WI61), equine-like strain THA/SKT-289, and the novel Venezuelan equine-like G3 VEN/3-CLC/2023.

A comparative in silico analysis of the VP7 gene sequences from Venezuelan equine-like strains revealed seven mismatches in the binding region of the G3 primer used in the second round of the G-typing reaction, three of them located around the six terminal bases at the 3′ end of the primer (Figure 1A).

On the other hand, the G9 primer used showed significant complementarity with the VP7 nucleotide sequence of the novel equine-like G3P[8] rotaviruses detected in 2023–2024, where 15 of 20 bases along the G9 primer were homologous (Figure 1B).

### 3.3. Phylogenetic Analysis of G3 Venezuelan Rotaviruses

**Figure 2:**
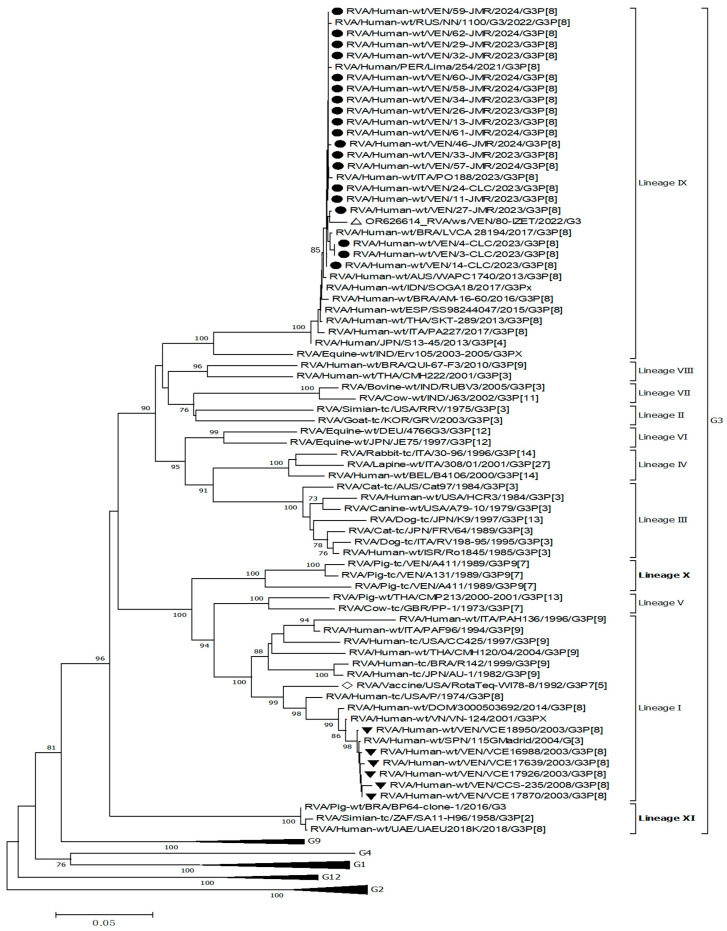
Phylogenetic tree of VP7 gene nucleotide sequences of Venezuelan human rotaviruses circulating from 2003 to 2024. The tree was generated by the neighbor-joining method and the Kimura two-parameter model as the substitution method. Bootstrap values above 70%, estimated with 1000 pseudo-replicate data sets, are indicated at each node. Venezuelan human G3 RVA isolated between 2003 and 2008 (old strains) [marked with a black down-pointing triangle (⯆)], equine-like G3 (novel G3) strains circulating in 2023–2024 [marked with a filled dot (●) symbol], and one wastewater G3 strain [VEN/80-IZET/2022, OR626614, marked by a white up-pointing triangle (Δ)], from Caracas, were compared to reference sequences from the GenBank database of the most common G1-4, -9, and -12 human and animal rotaviruses, and the RotaTeq^®^ vaccine G3 strain [marked with a white diamond (◊)]. VP7 non-G3 genotype clades were collapsed. Each taxon was named according to the proposed nomenclature of the Rotavirus Classification Working Group [57], and the rotavirus group, species isolated from, country of strain isolation, common name, year of isolation, and genotypes for genome segments 9 and 4 are indicated. The scale bar at the bottom of the tree represents genetic distance expressed as nucleotide substitutions/site.

Figure 2 shows the phylogenetic tree derived from the comparative analysis of VP7 gene nucleotide sequences from Venezuelan G3 rotaviruses. This analysis included 71 VP7 gene sequences of rotaviruses available in GenBank, including precisely 22 of human RVA across different G genotypes, and 49 from both human and animal G3. It revealed eleven different G3 lineages (I–XI) encompassing reported G3 strains, including porcine, simian, bovine, caprine, feline, equine, and human EQL-G3 strains, along with G3 ancestral strains.

Old and novel G3 Venezuelan VP7 genes were placed in distinct and well-supported branches compared to the reference strains. According to the G3 classification scheme previously described [33] and the results obtained in the present analysis, the six old 2003–2008 Venezuelan G3 strains grouped with previously reported G3 strains within lineage I, which included some ancient human G3 RVA, such as the strains P/1974, AU-1/1982, and CC4425/1997, altogether sharing 88.0–99.9% nt identity (Figure 2). The old Venezuelan strains were very similar to each other (99.3–100%) and formed a well-supported separate branch (bootstrap support 100%) together with some contemporary strains described elsewhere, such as 115GMadrid/2004, VN-124/2001, and 3000503692/2014 (98.7–99.9%). These old G3 Venezuelan RVAs shared 94.0–94.7% nt identity with the G3 strain of the WI78-8-RotaTeq vaccine strain.

The 19 novel 2023–2024 Venezuelan G3 strains formed a distinct, well-supported branch and demonstrated a nucleotide identity ranging from 99.3% to 100% with each other (Figure 2). These samples were grouped with representative strains of lineage IX (bootstrap support 99%), encompassing reported human equine-like-G3 strains (SKT-289 from Thailand and AM-16-60 from Brazil, among others) [29,35] along with the ancestral equine strain, Erv105/2003 (DQ981479), identified in India, with which all shared 90.7–99.9% nt identity. They exhibited the highest nucleotide identity with the human equine-like strains, PO188/2023, LVCA_28194/2017, NN/1100/2022, and Lima/254/2021 (99.3–99.9%), and were more distantly related to the older Venezuelan G3 rotaviruses (79.6% to 81% nt identity) and WI78-8-RotaTeq vaccine strain (80.2 to 80.8% nt identity).

The novel human 2023–2024 Venezuelan G3 strains also showed ≥98.9% nucleotide identity with the rotavirus G3 recovered from wastewater in Caracas in 2022 (80-IZET), which clustered into the same lineage IX.

Analysis of the VP7 gene from all G3 rotaviruses revealed two new previously undescribed discrete lineages of G3, which showed strong bootstrap values (=100). One of them included the Venezuelan porcine strains A131 (acc. n. L35055), A138 (L35079), and A411 (L35060) described from 1988 to 1989. These strains were grouped separately in a tentatively named lineage X, sharing 91.8–98.4% nt identity (Figure 2), and were differentiated from the other G3 lineages by percentages ranging from 11.9% to 21.3% (Table 2).

The other, tentatively named lineage XI, grouped the ancestral simian SA11 (LC178569), a porcine strain BRA/BP64-clone-1 (MN201922), and one human strain, UAEU2018K (ON500507), in a well-supported distinct branch (99.6–99.8% of intralineage nt identity, bootstrap 100%) (Figure 2). The nucleotide distance values of this new lineage XI compared to other lineages ranged between 15.4% and 20.6% (Table 2). To confirm these results and exclude clustering differences due to analysis artifacts, the Construct/Test Maximum Likelihood Tree tool from MEGA7 was applied.

### 3.4. Rotavirus Full Genome Analysis

To gain an insight into the genetic constellation of the EQL strains circulating in Caracas, a full-genome analysis of one representative strain 3-CLC detected in 2023 was performed. The nucleotide sequence analysis of all the gene segments revealed a genotype constellation including an equine-like G3P[8] in a DS-1-like backbone (I2–R2–C2–M2–A2–N2–T2–E2–H2). The analysis performed with the Rotavirus A Genotyping Tool, and BLAST searches confirmed the genotype assigned to the sequence of each single gene.

Following the proposed lineage classification for describing non-G and non-P genes of DS-1-like rotaviruses [58], the genomic constellation of the 3-CLC strain was I2-V, R2-V, C2-IVa, M2-VI, A2-IVa, N2-V, T2-V, E2-VI, H2-IVa.

**Figure 3:**
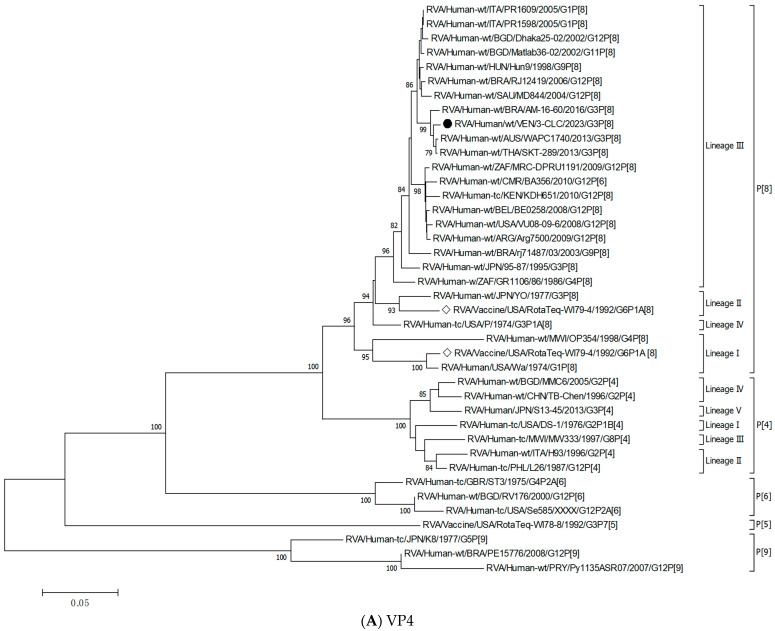

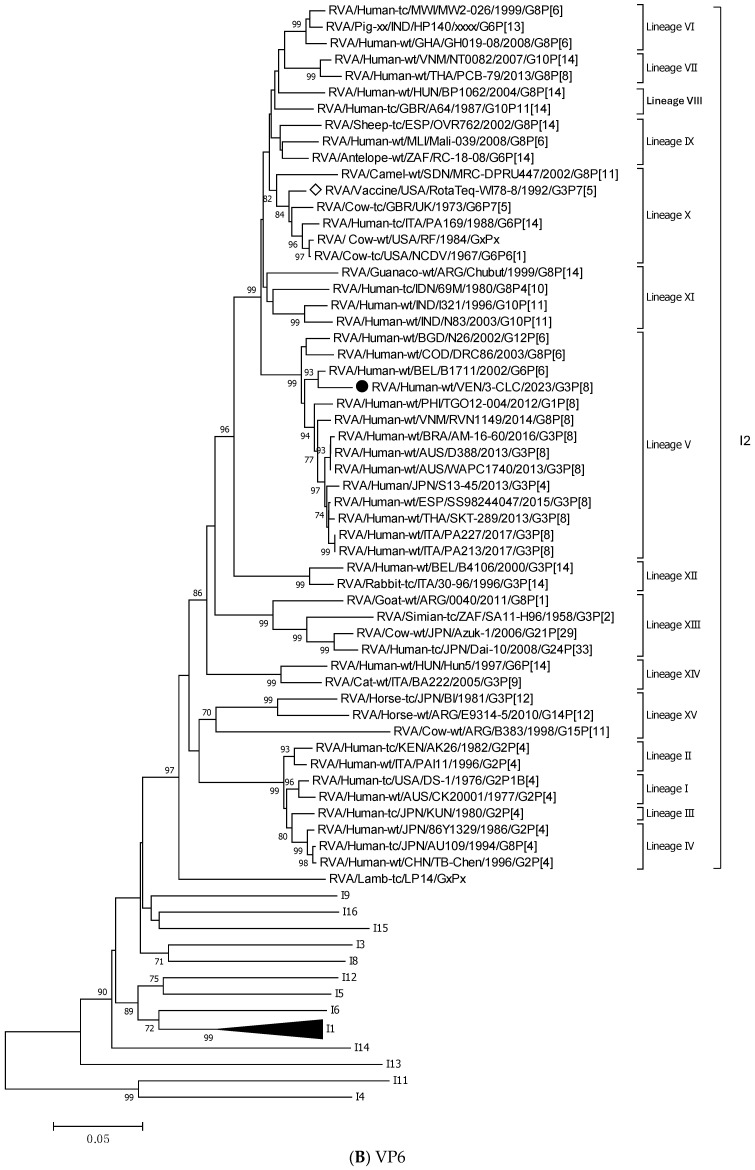

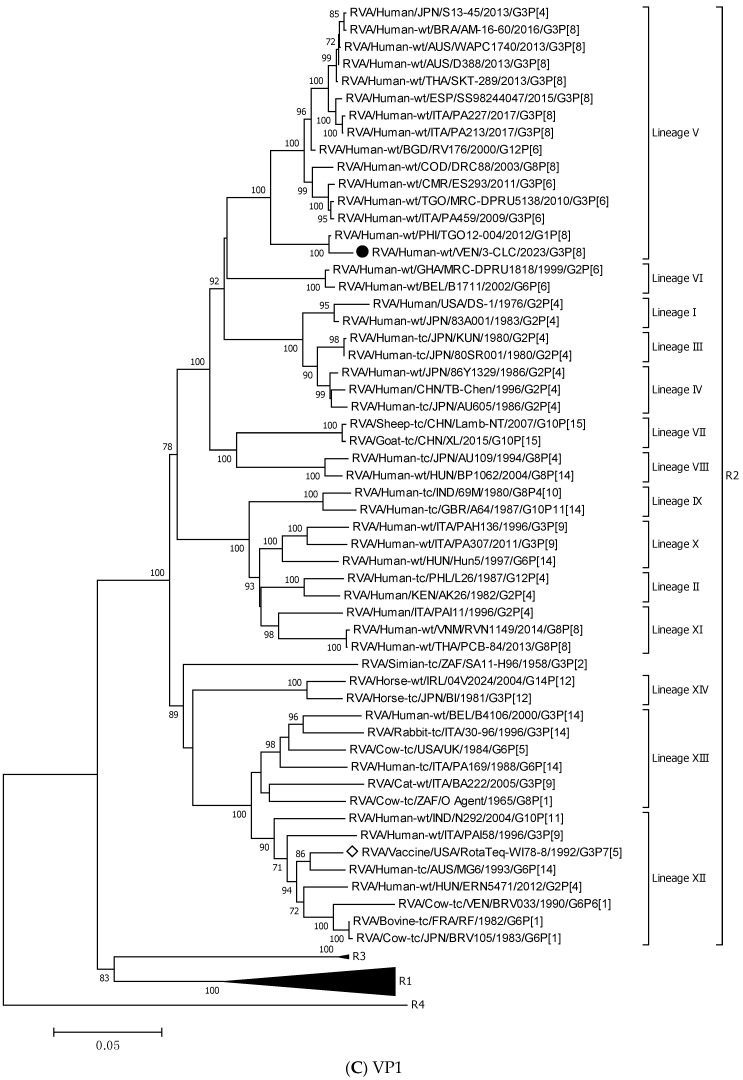

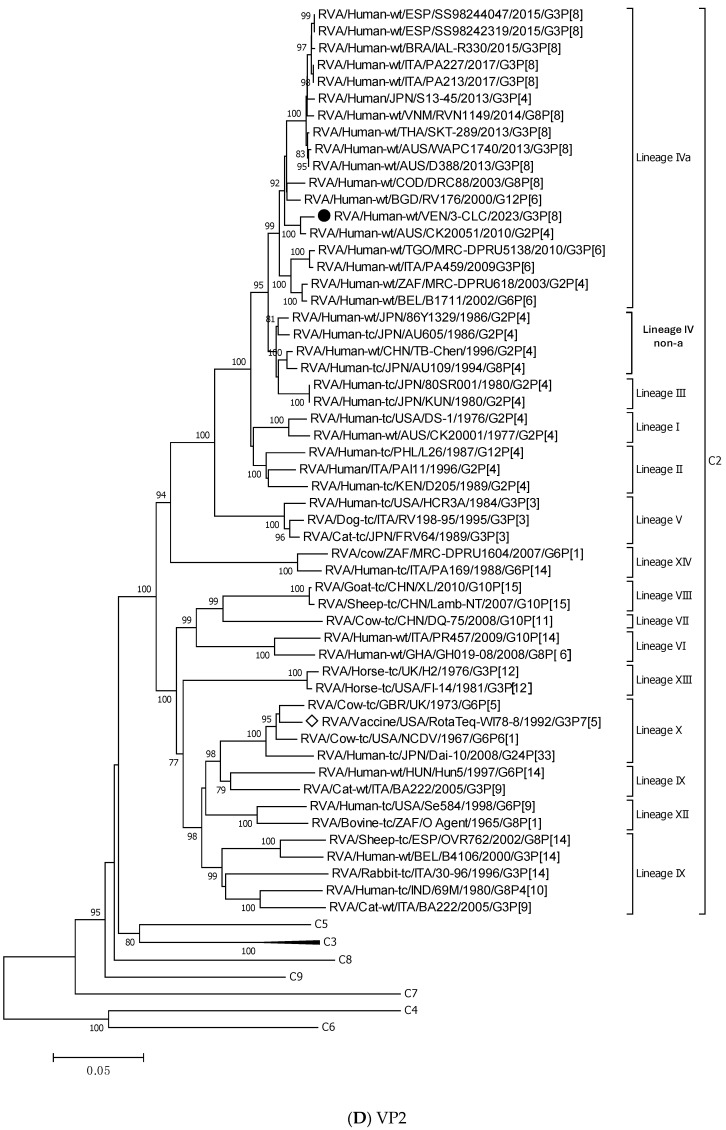

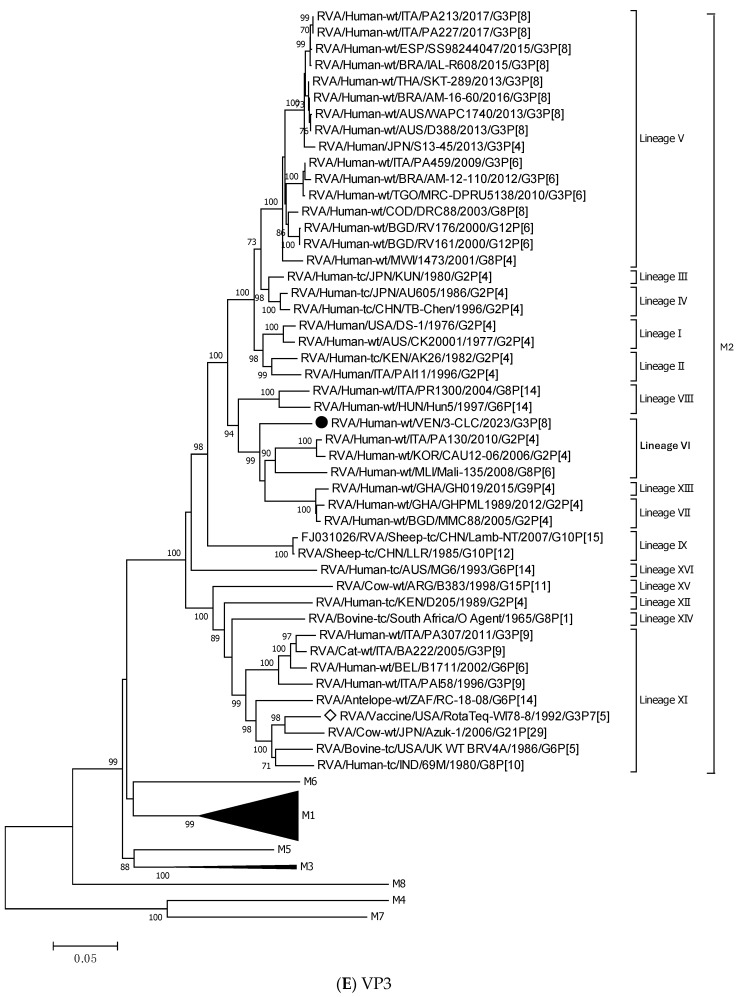

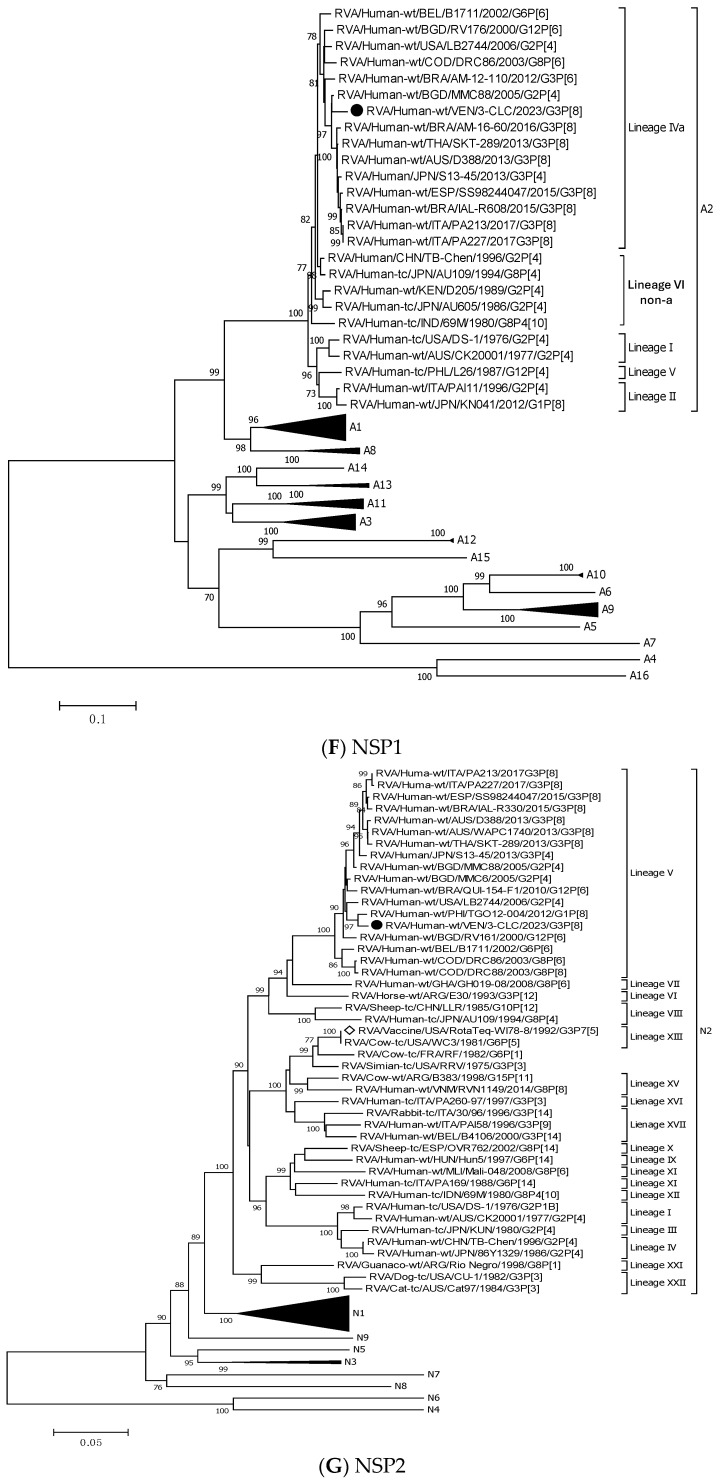

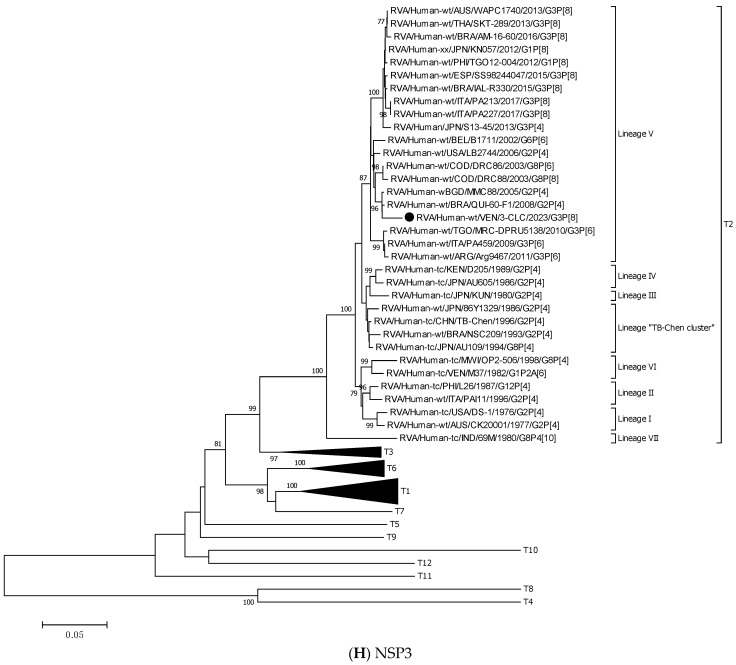

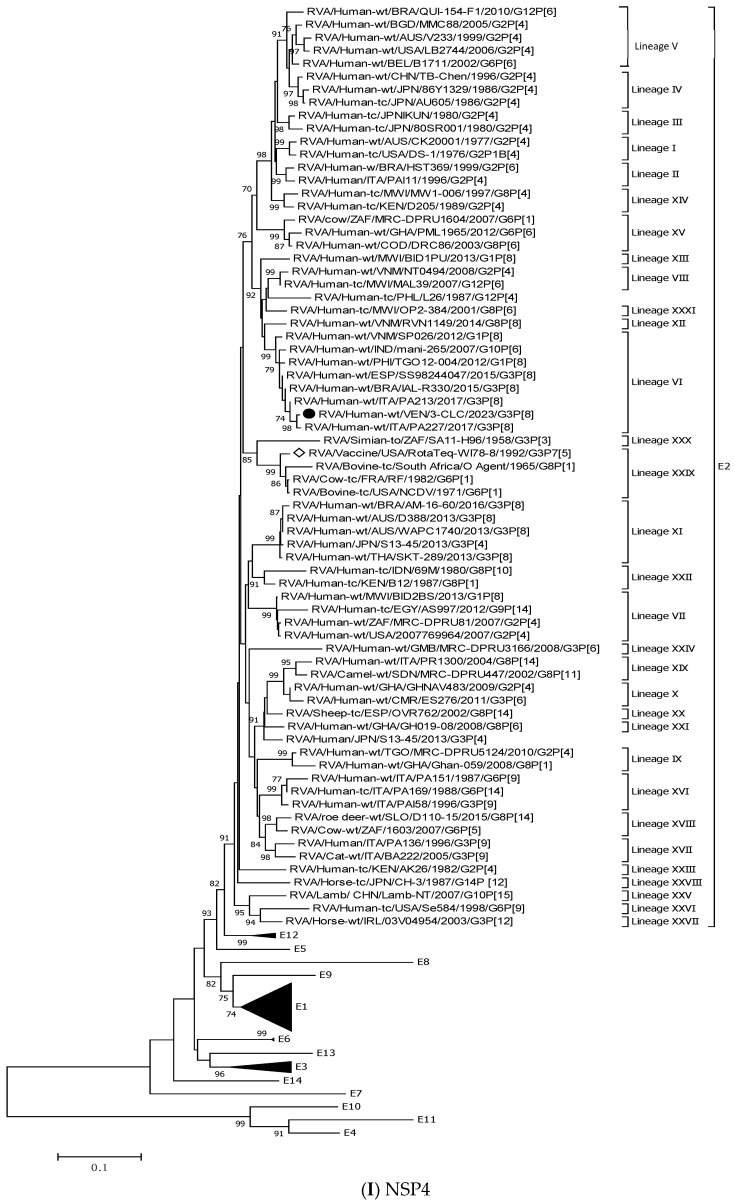

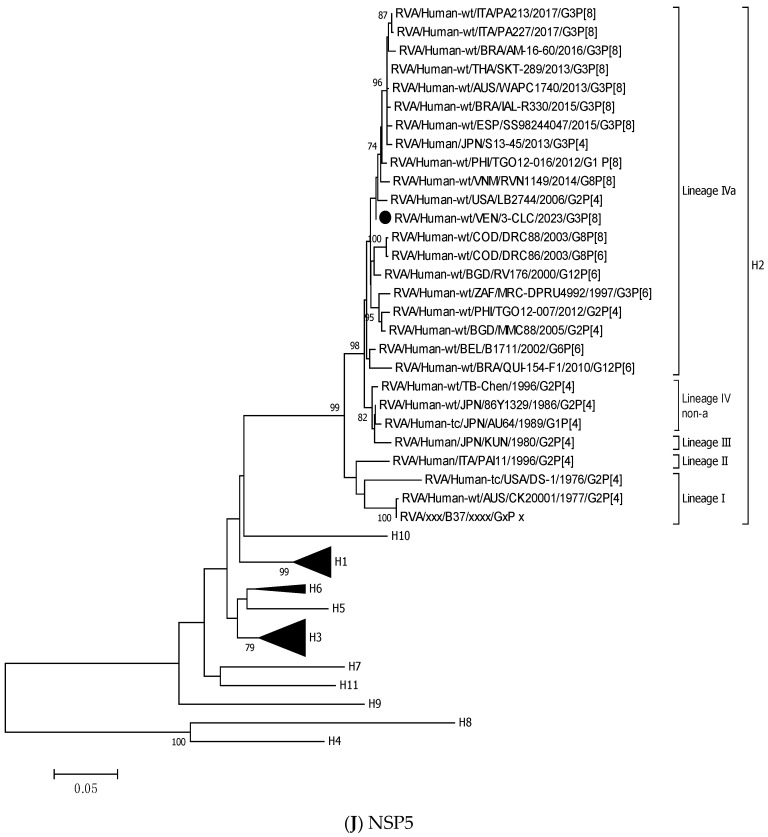
Phylogenetic trees constructed after full-genome analysis of one representative human equine-like G3P[8] rotavirus isolated in Venezuela in 2023. The nucleotide sequences obtained from all the rotavirus gene segments, VP4 (**A**), VP6 (**B**), VP1 (**C**), VP2 (**D**), VP3 (**E**), NSP1 (**F**), NSP2 (**G**), NSP3 (**H**), NSP4 (**I**), and NSP5 (**J**), from one human equine-like Venezuelan G3 collected in 2023, marked with a filled dot (●) symbol, were compared with reference strains available in GenBank. Gene sequences from RotaTeq™ and Rotarix^®^ vaccine strains, marked with a white diamond (◊), were included. Bootstrap values above 70%, estimated with 1000 pseudo-replicate data sets, are indicated at each node. The scale bar at the bottom of the tree represents genetic distance expressed as nucleotide substitutions/site.

For all the genes, the 3-CLC equine-like strain shared the same intratypic lineages with globally circulating equine-like G3P[8] strains, except for NSP4 and VP3 genes. Equine-like strains included in the analysis were segregated into two distinct sublineages of NSP4, E2-VI and E2-XI, both including human strains. The Venezuelan 3-CLC grouped into the lineage E2-VI and showed a close genetic relationship (99.3% nt identity) with human EQL strains from Brazil (IAL-R330/2015) and Europe (PA227/2017). The second cluster of NSP4 included equine-like prototypes of human rotaviruses from Thailand (SKT-289), Australia (WAPC1740), and Brazil (AM-16–60). On the other hand, the VP3 gene of 3-CLC clustered with human strains of the lineage VI of the M2 genotype, and it showed the highest identity (92.3%) with the strains ITA/PA130 and Mali-135 (Table 3, Figure 3E).

NSP5 and VP4 were among the more conserved genes, with intratypic nucleotide identities above 93.2% (Table 3). The 3-CLC demonstrated great closeness (>91.3%) to the ancestral strain DS-1 for VP2 and all the NSP genes, except NSP2 (Table 3).

The 3-CLC was closer to the P[8] equine-like strains SKT-289, WAPC1740, and AM-16-60 (of lineage P[8]-III) (98.1–99.3% of nt id) (Table 3) than to the ancient human P[8] strains of the same lineage III, such as JPN/95-87/1995 and Hun9/1998, and viruses of the ancestral lineage P[8]-I, such as the Wa strain, and OP354 (87.2–89.8% nt id) (Figure 3A) (Table 3).

Figure 3B displays that the VP6 gene of the 3-CLC study strain clustered more distantly with the ancestral DS-1 strain (nt identity 86.2%) (Table 3), but grouped close to equine-like G3P[8] strains that emerged in several countries from 2013 onwards (91.8–95.7%) and to the human reassortants G12 and G8P[6] with the DS-1 genomic constellation (Table 3). Both VP4 and VP6 genes of 3-CLC were among the genes that showed the highest similarity (92.2%) to the RotaTeq-WI78-8 vaccine strain.

On the other hand, the 3-CLC strain demonstrated the highest identity (99.7%) with the VP7 of the strain LVCA_28194 (Table 3), a human equine-like rotavirus isolated in Brazil in 2017 during a study in which similar EQL-G3 rotaviruses had become the predominant circulating genotype [59]. The VP1 gene of 3-CLC formed a well-supported cluster in lineage V with the human strain TGO12-004 (nt identity 98.8%), a human G1P[8] rearranged rotavirus with a DS-1-like genome constellation isolated in the Philippines (Table 3, Figure 3C). VP2 of 3-CLC clustered into the lineage VIa, close to the strain AUS/CK20051/2010 (nt identity of 99%) (Figure 3D). The NSP1 and NSP3 genes of the 3-CLC strain demonstrated a notable nucleotide identity (between 90% and 98.4%) with human strains of the same genotype, encompassing the ancestral DS-1 (nt id 91.6% and 94.1%, respectively, for A2 and T2 type) (Table 3), and human equine-like strains, whose nt identities were among the highest (96.2–97.5%) (Table 3, Figure 3F,H). NSP2 analysis grouped the 3-CLC strain into the N2 genotype together with the ancestral DS-1 strain (which was moderately distant, 86.9%), and close to viruses of different animal origins (canine, feline, lapine, bovine, ovine, and human). It clustered mainly close to human equine-like strains (96.5–97.15 of nt id) but distant from the RotaTeq-WI78-8 vaccine strain (87.2% nt id) (Table 3, Figure 3G).

### 3.5. Comparison of Deduced VP7 Amino Acid Sequences

The alignment comparison by MEGA of the 25 deduced amino acid VP7 sequences from the Venezuelan G3P[8] strains with the WI78-8-RotaTeq™ vaccine strain belonging to the lineage G3-I and one representative human equine-like strain (SKT-289) revealed that the novel strains from Caracas shared an amino acid identity ≥ 98.9% with representative strains of lineage IX (≥99.1%, encompassing several reported human equine-like G3 strains.

The amino acid distance between the novel Venezuelan G3 rotaviruses (2023–2024) and the VP7 from older Venezuelan G3 strains (2003–2008) ranged from 7.7 to 8.9%. When compared with the WI78-8-RotaTeq™ vaccine strain, this distance was between 7.2 and 7.9%, which was higher than that observed with the older G3P[8] strains circulating in Venezuela between 2003 and 2008 (2.8–3.0%).

The analysis of synonymous and non-synonymous substitutions for 180 codons of the VP7 protein by Highlighter and SNAP tools showed that the novel Venezuelan G3 rotaviruses (2023–2024) sequences do cluster according to a similar mutation pattern. There were significantly more synonymous substitutions than non-synonymous ones, suggesting an overall phenotypic stability in the VP7 glycoprotein of these novel G3P[8] strains. The ratio between synonymous (ds) and non-synonymous (dn) substitutions was well above 1 (dS/dN >> 1). Specifically, the averages of all pairwise comparisons of the VP7 sequences were ds = 0.8707, dn = 0.0127, ds/dn = 26.7429, ps/pn = 9.9895, and the averages of the comparison with the WI78-8-RotaTeq™ vaccine strain were ds = 3.0325, dn = 0.0387, ds/dn = 78.4760, and ps/pn = 19.5447.

**Figure 4:**
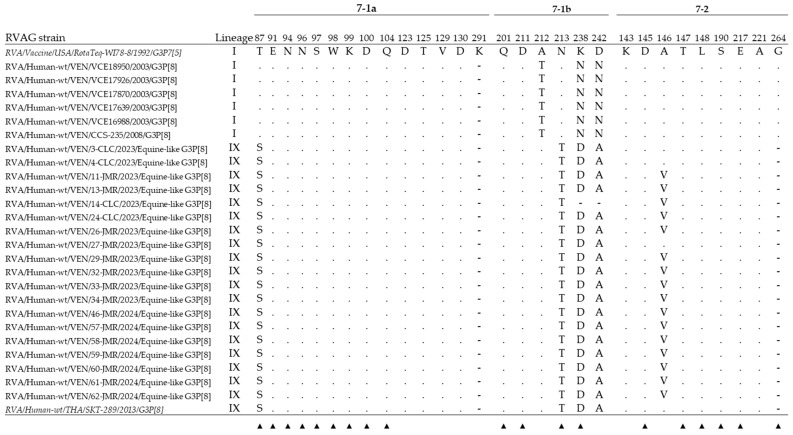
Alignment of the antigenic residues from deduced amino acid sequences defining the neutralization domains on the VP7 protein of the rotaviruses analyzed: Neutralization domains of VP7 are designated as 7-1a, 7-1b, and 7-2 [60]. Deduced amino acid sequences ranging from 180 to 284 residues obtained from Venezuelan G3P[8] strains were compared with the WI78-8-RotaTeq^®^ vaccine strain (GU565079) of lineage G3-I, and the reference human equine-like SKT-289 (LC086730) from Thailand of lineage IX (both in italics). For each strain, host species, country of origin, year of isolation, and genotypes G-P are shown. Numbering is based on the open reading frame of WI78-8-RotaTeq^®^ vaccine strain. The sites not included in the analysis are indicated with a hyphen (-). In each isolate, identical amino acids with the RotaTeq™ vaccine strain are identified by dots. Amino acid changes that have been shown to escape neutralization with monoclonal antibodies [60] are indicated with a filled triangle (▲).

Thirteen amino acid changes were visualized in the deduced amino acid VP7 sequences from the novel Venezuelan G3 RVAs in comparison with the WI78-8-RotaTeq^®^ vaccine strain. The analysis of the residues that define the neutralization domains of capsid proteins VP7, designated as 7-1a, 7-1b, and 7-2 [60], revealed that these strains shared the same amino acid substitutions on the VP7 as the human equine-like G3 strain SKT-289, which belongs to the same lineage IX (Figure 4). The main amino acid changes, predominantly identified within the VP7 neutralization epitopes, were T87S, A146V, N213T, K238D, and D242A (Figure 4), three of them situated in escape neutralization sites within the hypervariable regions of the VP7 protein, as demonstrated by monoclonal antibody studies (Figure 4) [60].

## 4. Discussion

The RVA infection rate observed in children with diarrhea from Caracas during 2023–2024 following the COVID-19 pandemic was relatively higher than that found between 2003 and 2013 in different cities in Venezuela (Table 1) [46,47,53]. The predominance of the G3P[8] genotype is noteworthy, given that human G3 rotaviruses had not been detected in Venezuela since 2008 [47].

The VP7 phylogenetic analysis of all the rotavirus strains detected in 2023–2024, which were untypable in the multiplex PCR assay and carried a VP4 of the P[8] type, confirmed their similarity with G3 strains and a high identity with each other. They collectively shared high genetic similarity with representative strains of lineage IX of G3, which includes described human equine-like strains along with the ancestral equine strain, emerging globally from 2013 onwards [10,27,31,32,33,59].

The failed G-typing of the novel G3 equine-like Venezuelan strains can be attributed to the discrepancies of sequence observed with the G3 primer, particularly at the 3′ end. Additionally, the mis-priming of the G9 primer, possibly due to sequence similarities or sub-optimal annealing temperatures, may have resulted in the incorrect genotype assignment of some equine-like G3 samples as G9. This issue has been documented in previous studies [61]. Validation performed with a pair of specific equine-like G3 VP7 primers emphasizes the need for ongoing revision and updates of the type-specific primers used in the genotyping protocols, as natural variations in the VP7 and VP4 genes can impact the accuracy of the tests [27,31,32].

G3P[8] strains identified in this study differed by over 19% from old G3 rotaviruses circulating in Venezuela before 2008, which were closer to ancient Venezuelan porcine strains than the novel human equine-like G3 [22]. Thus, the G3 genotype has likely undergone significant divergence over time on a global scale, resulting in the introduction to the country of a virus that is increasingly distanced from the porcine species, but still of animal origin.

The high genetic similarity (over 99%) between the novel equine-like G3P[8] strains and the rotavirus detected in wastewater in 2022 [48] implies that equine-like strains have been circulating before this study, highlighting the infectious potential of G3 [33].

Equine-like G3P[8] rotaviruses have been identified in children from several countries, such as Australia, Japan, Thailand, Italy, Spain, Haiti, and the Dominican Republic [27,32,33,34]. In Brazil, a neighboring country of Venezuela, equine-like G3P[8] has rapidly spread since 2015 and displayed a marked potential to replace Wa-like G3P[8] strains [36]. Their emergence in Venezuela corroborates the widespread dissemination in Latin America and confirms that these strains have acquired biological properties that make them more transmissible.

In this study, the phylogenetic analysis of VP7 gene sequences from G3 reference rotaviruses displayed a wide intratypic genetic heterogeneity, possibly due to the greater selective immune pressure to which VP7 is subjected. The distance matrix analyses resulted in one preliminary identification of two new lineages (X and XI), for strains that were otherwise unclassifiable, grouped in clusters with well-supported branches within genotype G3. Although a more comprehensive analysis would be desirable, the proposed lineage definition for G3 provides a valuable contribution, enhancing the understanding of its genetic heterogeneity.

Full-genome analyses of one representative strain, named 3-CLC, aimed to investigate the relationship of the novel Venezuelan G3 rotaviruses with other similar equine-like G3s. The molecular characterization of the 11 gene segments of the strain 3-CLC revealed that it contained an equine-like G3 VP7 gene and a P[8] VP4 gene, with an I2-R2-C2-M2-A2-N2-T2-E2-H2 backbone. Such a genomic constellation has been reported to derive from reassortment event(s) between strains with a DS-1-like genome from humans with human and/or animal rotaviruses [27]. Because the DS1-like strains’ genes are very well adapted to infect humans, the presence of an equine-like VP7 (G3) gene, along with a P[8], the most common VP4 type among the human rotaviruses, could make the novel Venezuelan rotaviruses very well equipped to become a predominant and important pathogen in this country.

The 3-CLC strain demonstrated a high genetic similarity with the globally prevalent equine-like G3P[8] strains for most of the genes, which agrees with the expansion of a highly conserved genome and the existence of a common ancestor. It is plausible that the transmission of these strains from animals arose some time ago, possibly in another geographical context, and that they have been present and circulating within the human population for an extended period, as suggested by studies conducted in geographically close countries [34,59]. On the other hand, the segregation of the equine-like strains in two lineages of the E2 type of the NSP4 gene, lineage E2-V and lineage E2-XI, supports the previously described hypothesis of the existence of two different co-circulating equine-like clades in the world [36]. The E2-VI lineage carried by the Venezuelan strain 3-CLC is different from that reported for the prototype equine-like G3P[8] strains detected in Australia (WAPC1740/2013/G3P[8]), which was speculated to be of animal RVA origin [27]. The segregation of the Venezuelan 3-CLC strain into VP3 M2 lineage VI, distinct from the globally circulating equine-like strain, suggests an upcoming diversification of these strains.

The significant deduced amino acid sequence differences between these emerging strains and the currently used rotavirus vaccines raise concerns about their efficacy. The VP7 of the novel equine-like G3 strains described in the present study showed a low genetic similarity with the WI78-8-RotaTeq vaccine G3 strain, one of the two vaccines used in Venezuela until recently, carrying the same genotype. SNAP analysis shows that observed synonymous mutations were the predominant class of mutations that could have been fixed within the VP7 of equine-like G3P[8] strains. This suggests that despite the pressure of the immune system, the VP7 shows overall phenotypic stability across the equine-like G3 genotype, aside from a few highly variable regions. The results confirm that adaptative evolution was not an important driving force during the viral spread of these strains in humans [59].

On the other hand, it is known that mutations in crucial amino acid residues defining neutralization domains of the capsid protein VP7 may influence the effectiveness of rotavirus vaccines [62], which are more effective against homotypic strains than partially or fully heterotypic strains [63]. All the novel equine-like strains circulating in Venezuela showed several antigenic changes in the neutralization epitopes 7-1a, 7-1b, and 7-2 located on the variable regions of the VP7 protein, and at least three of them (T87S, N213T, and K238D) appear to be conserved among most equine-like G3 strains described [27,32,35]. These changes arouse interest, particularly the K238D substitution, which implied a radical shift from a positively (lysine) to a negatively (aspartic acid) charged amino acid, located in defined escape neutralization sites [60], along the surface-exposed antigenic epitopes on the VP7. Such changes in the surface charge may determine an altered conformational structure of some antigenic regions on the outer capsid viral protein. Furthermore, the N213T mutation determines the disappearance of a potential N-linked glycosylation site, whose effect is unknown. It has been demonstrated that differences in the N-linked glycosylation of proteins can alter their immunogenicity and directly affect antibody-mediated neutralization [64]. The accumulation of these amino acid changes could have contributed to selecting one rotavirus variant with enhanced transmissibility among humans. Thus, combined with a potential decline in specific protective immunity within the population, they may have acted synergistically, leading to the predominance of G3 strains in recent years. Further studies are required to understand this matter and its impact on vaccine efficacy.

Equine-like G3P[8] and G2P[4] strains have been described to be predominant in areas where the Rotarix^®^ vaccine has been used [44]. In the local context, the use of both Rotarix^®^ and RotaTeq^®^ has decreased significantly in recent years. They are primarily available in the private market, limiting access. Thus, it is unlikely that the immunization program could have contributed to the emergence of rotavirus vaccine escape strains in Venezuela. On the other hand, a study conducted in Haiti demonstrated that the Rotarix^®^ monovalent vaccine was effective in preventing hospitalizations due to the equine-like G3P[8] strain, emphasizing the importance of ensuring a complete and timely vaccination to protect against rotavirus [65]. The recent significant proportion of equine-like G3 rotavirus-infected children underscores the need to monitor the vaccine impact on the emergence of escape mutants or the potential spread of unusual zoonotic strains that could drive global epidemic patterns.

This study might have a bias in the rotavirus detection rate due to the sampling method used, which included a limited number of children, aged up to 12 years, unlike our previous studies. The seasonality of infection could not be assessed due to the challenges in obtaining samples regularly over time, as logistical viability and resource availability affected the selection procedure. Because the analysis included only a small portion of the children affected, it does not accurately represent the entire population and therefore has little statistical power. Although the evidence points to the G3 being predominant, the limited sampling may have hidden the detection of other rotavirus genotypes during the study period. Whether equine-like G3 strains are disseminated throughout the nation is likewise unknown. Nevertheless, the genetic analysis of the strains obtained remains valuable as they can be combined in future meta-analyses.

## 5. Conclusions

The unusual dominance of an emerging human equine-like G3 rotavirus following the COVID-19 pandemic raises significant concerns, particularly regarding the potential reduced effectiveness of currently used rotavirus vaccines against it.

Although temporal and regional fluctuations in rotavirus genotype distribution appear to occur independently of vaccine implementation, the appearance of variants in the post-vaccine era should be analyzed thoroughly to assess the genetic context associated with their emergence. The changes observed in antigenic determinants of the outer capsid proteins, such as the VP7, suggest a potential ability of these viruses to evade vaccine protection, contributing to their national and global dissemination.

Continued epidemiological and genetic data will be essential for understanding how rotaviruses will continue evolving during and between epidemic seasons. The results highlight the importance of implementing preventive measures such as vaccination, whose efficacy should be unceasingly revised to guarantee their success and adapt molecular surveillance methods to the changes that arise as viruses evolve.

## Figures and Tables

**Table 1:** Historical human G3 rotavirus strains from children with diarrhea detected in Venezuela between 2003 and 2013.

Period	City	Total n. Samples Studied	No. Rotavirus-Positive Samples (%)	No. G3 Identified (%)	No. Samples Analyzed in This Study	References
2003	Valencia	480	102 (21.0)	46 (52.9) *	5	[46]
2007–2008	Caracas	912	206 (22.6)	1 (0.6) *	1	[47]
2011–2013	Maracay	213	25 (11.7)	0	-	Personal communication
2012–2013	Barcelona	150	27 (18.0)	0	-	[53]

* Based on multiplexed RT-PCR G-typed strains.

**Table 2:** Intralineage and interlineage nucleotide identity of the VP7 gene among the G3 strains analyzed in this study.

	Nucleotide Identity ^(^*^)^	
Lineage G3	% Intralineage	% Interlineage
I	88–100	76.4–89.5
II	89.1	79.6–88.9
III	94.2–98.8	76.6–89.6
IV	95.6–95.8	76.6–89.6
V	93.9	80.0–89.5
VI	93.4	79.0–89.5
VII	97.3	78.3–87.7
VIII	91.7	78.7–88.7
IX	90.7–100	78.0–88.9
X ^(^**^)^	91.8–98.4	78.7–88.1
XI ^(^**^)^	99.6–99.8	79.4–84.6

* The nucleotide identities were based on distance matrices prepared using the p-distance algorithm in MEGA7, and 1000 replicates to estimate branch support. ** Revised to be proposed as a new lineage in this study.

**Table 3:** Genotype constellation of one representative G3P[8] equine-like rotavirus strain (sample 3-CLC) isolated in Caracas, Venezuela, in 2023 and comparison with other rotavirus strains.

Gene	Genotype Constellation	Lineage	Most Similar Strain	Intratypic % Nt Identity *	Intertypic % Nt Identity *	% Nt Identity vs. EQL−Strains **	% Nt Identity vs. Vaccine Strain ^	% Nt Identity vs. Ancestral Prototype Strain #
VP7	G3	IX	RVA/Human−wt/BRA/LVCA_28194/2017/G3P[8] »	78.9–99.7	70.5–78.9	**99.0–99.5**	80.8 (75.3)	81.7
VP4	P[8]	III	RVA/Human−wt/AUS/WAPC1740/2013/G3P[8] »	**95.5–99.3**	58.6–86.1	**98.1–99.3**	**92.2** (89.2)	89.8
			RVA/Human−wt/THA/SKT−289/2013/G3P[8] »					
VP1	R2	V	RVA/Human−wt/PHI/TGO12−004/2012/G1P[8]	85.8–98.8	71.5–80.8	86.3–93.3	86.4 (80)	88.6
VP2	C2	IVa	RVA/Human−wt/AUS/CK20051/2010/G2P[4]	84.6–99.0	69.7–82.7	**96.6–97.1**	85.2 (81.3)	**94.7**
VP3	M2	VI	RVA/Human−wt/ITA/PA130/2010/G2P[4]	81.5–92.3	62.5–77.1	87.7–88.8	81.5 (75.5)	89.9
			RVA/Human−wt/MLI/Mali−135/2008/G8P[6]					
VP6	I2	V	RVA/Human−wt/BEL/B1711/2002/G6P[6]	82.8–97.8	68.6–92.2	**91.8–95.7**	**92.2** (78.5)	86.2
NSP1	A2	IVa	RVA/Human−wt/BGD/MMC88/2005/G2P[4]	**90.0–98.1**	39.8–75.1	**96.2–97.5**	67.6 (74.5)	**91.6**
NSP2	N2	V	RVA/Human−wt/PHI/TGO12−004/2012/G1P[8]	82.4–98.8	60.9–83.7	**96.5–97.1**	87.2 (82.9)	86.9
NSP3	T2	V	RVA/Human−wt/BGD/MMC88/2005/G2P[4]	**90.3–98.4**	53.7–84.7	**96.2–96.3**	77.3 (78.3)	**94.1**
NSP4	E2	VI	RVA/Human−wt/ITA/PA227/2017/G3P[8] »	85.5–99.3	50.8–86.4	87.7–99.3	88.0 (80.3)	**90.1**
			RVA/Human−wt/BRA/IAL−R330/2015/G3P8					
NSP5/6	H2	IVa	RVA/Human−wt/THA/SKT−289/2013/G3P[8] »	**93.2–99.2**	59–85.7	**98.7–99.2**	84.9 (83.3)	**93.2**
			RVA/Human−wt/PHI/TGO12−016/2012/G1P[8]					

In bold, significantly high values of nt identity (>90%). nt = nucleotide. (*) Comparison between the representative EQL G3 Venezuelan 3-CLC strain and reference strains used for the full-genome analysis. (**) Included human EQL-G3 strains that clustered with EQL-G3P[8] prototype strain in a clade with well-supported bootstrap results. (^) The rotavirus vaccine strains used for the comparison were the RotaTeq-WI78-8/1992/G3P[5] and the Rotarix-A41CB052A/1988/G1P[8] (between parenthesis) for all genes except VP4 for which RotaTeq-WI79-4/1992/G6P[8] was used. (#) The ancestral prototypes were the strains “P” for G3, “Wa” for P[8], and “DS-1” for the remaining genes. (») Human equine-like strains reported elsewhere.

## Data Availability

The data that support the results are available from the corresponding author upon reasonable request. The authors have confirmed that the nucleotide sequence data for the rotaviruses analyzed in this study have been submitted to the GenBank nucleotide sequence database (under the accession numbers PV036887-PV036921).

## Supplementary Materials

The following supporting information can be downloaded at https://www.mdpi.com/article/10.3390/v17030410/s1: Table S1: Oligonucleotide primers used for the full genome analysis of rotavirus in this study [66].
