# Supplementary material for: Emergence of Equine-like G3P[8] Rotavirus Strains Infecting Children in Venezuela
Source: Viruses. 2025 Mar 13;17(3):410. doi: 10.3390/v17030410 (PMC11946648; doi:10.3390/v17030410)
Supplement: Supplementary file 1 [file viruses-17-00410-s001.zip › viruses-3480262-supplementary.pdf]

Table S1: Oligonucleotide primers used for the full genome analysis of rotavirus in this study.

Gene segment	Primer name	Sense	Primer sequence 5´ - 3´	Nucleotide position	Amplicon size (bp)	Reference
VP1	GEN_VP1 Fb	+	GGC TAT TAA AGC TRT ACA ATG GGG AAG	1-27		[24]
	VP1-RV1 *	-	CCA TTA GCC ATR TCA TCC AT	1333-1352	1352	In this study
VP2	VP2-F1D *	+	GTC ATT RAT TTC AGG HAT GTG G	1219-1240	1419	In this study
	GEN_VP2_Rbc	-	GTC ATA TCT CCA CAR TGG GGT TGG	2660-2683		[24]
VP3	GEN_VP3Fe	+	GGC TWT TAA AGC ART ATT AGT AGT	1-24	1271	[24]
	VP3-R1D*	-	CTG AYC TAA TYT CYG TAG TTG G	1250-1271		In this study
VP4	A	+	TGG CTT CGT TCA TTT ATA GAC A	11-32	1083	[51]
	B	-	CTA AAT GCT TTT GAA TCA TCC CA	1072-1094		[51]
VP6	GEN-VP6-F	+	GGC TTT WAA ACG AAG TCT TC	1-20	1356	[24]
	GEN-VP6-R	-	GGT CAC ATC CTC TCA CT	1340-1356		[24]
VP7	VP7-F	+	ATG TAT GGT ATT GAA TAT ACC AC	49-71	884	[50]
	VP7-R	-	AAC TTG CCA CCA TTT TTT CC	914-933		[50]
NSP1	GEN_NSP1F	+	GGC TTT TTT TTA TGA AAA GTC TTG	1-24	1554	[24]
	GEN_NSP1R	-	GGT CAC ATT TTA TGC TGC C	1544-1564		[24]
NSP2	MAX-NSP2F	+	GGC TTT TAA AGC GTC TCA GTC	1-21	1059	[24]
	MAX-NSP2R	-	GGT CAC ATA AGC GCT TTC TAT	1039-1059		[24]
NSP3	MAX-NSP3F	+	GGC TTT TAA TGC TTT TCA GTG	1-21	1064	[24]
	MAX-NSP3R	-	GGT CAC ATA ACG CCC CTA TAG	1044-1064		[24]
NSP4	NSP4 SE	+	GGC TTT TAA AAG TTC TGT TCC GAG	1-24	750	[66]
	NSP4 AS	-	GGT CAC ACT AAG ACC ATT CC	731-750		[66]
NSP5	MAX-11F	+	GGC TTT AAA GCG CTA CAG TGA	2-22	666	[24]
	MAX-11R	-	GGT CAC AAA ACG GGA GTG GGG	647-667		[24]

*These primers were based on the reference strain DS-1 genome

Degenerate primers; R=A/G, W=A/T, Y=C/T
